# Cognitive Intervention with Musical Stimuli Using Digital Devices on Mild Cognitive Impairment: A Pilot Study

**DOI:** 10.3390/healthcare8010045

**Published:** 2020-02-25

**Authors:** Eunyoung Han, Jinse Park, Haeyu Kim, Geunyeol Jo, Hwan-Kwon Do, Byung In Lee

**Affiliations:** 1 Department of Music Therapy, Graduate School, Ewha Womans University, Seoul 52, Korea; heyewha@gmail.com; 2 Department of Neurology, Haeundae Paik Hospital, Inje University, Busan 50834, Korea; BILEE@paik.ac.kr; 3 Department of Neurological Surgery, Haeundae Paik Hospital, Inje University, Busan 50834, Korea; hykim080356@gmail.com; 4 Department of Physical Medicine and Rehabilitation, Haeundae Paik Hospital, Inje University, Busan 50834, Korea; hprm@paik.ac.kr (G.J.); satirev83@gmail.com (H.-K.D.)

**Keywords:** mild cognitive impairment, music therapy, executive function, technology

## Abstract

The effect of music therapy on cognitive function has been widely reported; however, its clinical implications remain controversial. Performing therapeutic musical activities in groups using individualized instruments can help overcome the issues of engagement and compliance. We aimed to evaluate the effect of a cognitive intervention with musical stimuli using digital devices on mild cognitive impairment (MCI). In this prospective study, 24 patients with MCI (intervention group, 12; and control group, 12) were enrolled. We developed an electronic device with musical instruments and the Song-based Cognitive Stimulation Therapy protocol (SongCST). Patients in the intervention group underwent a 10-week cognitive intervention involving musical stimuli generated by our device. Effect of the intervention on cognitive function was evaluated by the Mini-Mental State Examination-Dementia Screening (MMSE-DS), Montreal Cognitive Assessment-Korean (MOCA-K), and Clinical Dementia Rating Scale Sum of Boxes (CDR-SOB). In the intervention group, MMSE-DS and MOCA-K scores improved significantly after the 10-week intervention. The changes in MOCA-K and CDR-SB scores were significantly different between the intervention and control groups. Our study showed that music therapy with digital devices has a positive effect on the executive function and overall disease severity in patients with MCI. Our study can facilitate individualization of music therapy using digital devices in groups.

## 1. Introduction

With an increase in the global elderly population, the incidence of dementia has also increased. Alzheimer’s disease (AD) is the most common neurodegenerative disease, and it has been a topic of interest in the last few decades [1]. Unfortunately, none of the drugs including acetylcholine esterase inhibitors (AchEi) and NMDA antagonists have been able to cure the condition. The pharmacological treatment can only delay the disease progression to the stage of disability for 2 years [2]. Because of repetitive failure of clinical trials with drugs targeting amyloid β and tau protein for dementia, non-pharmacological treatment has been emerging [3,4]. Numerous forms of non-pharmacological treatments to improve the cognitive function have been investigated, but their effects on dementia remain controversial [5].

Mild cognitive impairment (MCI) marks the preclinical stage of AD, and the conversion ratio to AD is up to 80% within 5 years [6]. MCI is the target stage of pharmacological and non-pharmacological therapies for preventing progression to AD. A meta-analysis reported the presence of moderate-quality evidence suggesting that cognitive training (CT) and cognitive stimulation show a small to moderate effect on early dementia and MCI [7]. CT, which involves repetitive training in a specific domain, showed a positive effect on dementia and MCI [4]. Cognitive stimulation, which involves using enjoyable entertainment activities for cognitive intervention, also showed a positive effect on the early stage of dementia and MCI [8]. Verghese et al. demonstrated that four leisure activities including reading, playing board games, dancing, and playing musical instruments significantly reduced the risk of dementia among various leisure activities evaluated [9].

Despite showing positive results, non-pharmacological treatment is not widely used in clinical settings because of cost ineffectiveness and problems with adherence [10]. Most studies were performed under well-controlled environments with enough financial support; therefore, there have been few reports showing the long-term effects of non-pharmacological treatment [7]. Consequently, home-based training sessions or hands-on activities for patients have been introduced as more effective interventions than community-based cognitive interventions. Recently, a fluorodeoxyglucose (FDG)-positron emission tomography (PET) study showed that individual home-based CT using a pen and home-work sheets possibly increases cognitive function and shows a positive effect on the metabolic activities occurring in the brain [11]. For the popularization of home-based training, it is necessary to develop individual intervention with low cost and personalized training program with enjoyable contents. 

Among various non-pharmacological interventions, music therapy has attracted much interest regarding cognitive stimulation in patients with cognitive decline [12]. Numerous studies have demonstrated the efficacy of music therapy in improving the cognitive function in patients with AD [13]. The effect of music therapy in patients with MCI has also been reported [14]. Especially, playing musical instruments significantly improved the frontal lobe function in patients with MCI [15]. Recently, focusing on multitask movements such as eye-hand coordination and auditory-motor synchronization in music therapy has demonstrated an increase in frontal lobe function using neuropsychological tests and imaging techniques [16]. Reportedly, dual musical tasks activate the frontal lobe function and attentiveness in elderly patients.

We developed a cognitive intervention involving musical stimuli based on therapeutic mechanisms of human rhythmicity, administered using digital devices [17] . Human rhythmicity implies inherent rhythms of the human body (e.g., breath, heart, walking, running) and learned connections between these and musical properties according to the four-level model of mechanisms of musical responses [18]. Our therapeutic intervention involved composing cognitive musical stimulation for engaging synchronized hand movement with external auditory-visual cues using digital instruments.

We aimed to engage patients with innovative therapeutic musical stimuli and activities that are intensive and individualized through cognitive stimulation using multidisciplinary approaches. The purpose of our study was to investigate the effect of our cognitive intervention with musical stimuli using digital devices in patients with MCI.

## 2. Methods

### 2.1. Study Design and Subjects

This prospective, open-label, randomized, case-controlled, pilot trial was conducted for a duration of 12 weeks (pre-post test included; sessions for 10 weeks, 2 times/week, 60 minutes/session) with a music therapist. According to a previous study, administration of cognitive stimulation therapy (CST) once a week (45 minutes) for 14 weeks did not show any significant effect [19]. It was suggested that a more frequent administration of intervention is needed. Therefore, sessions were conducted twice a week for 10 weeks (20 sessions) considering public elderly program schedules.

This study included patients who visited a clinic in Haeundae Paik hospital via the Gi-Jang public dementia care center. Inclusion criteria were as follows: (1) diagnosis of MCI according to clinical criteria [20]; (2) age of 60–90 years; and (3) an understanding of and the ability to manipulate the device. Exclusion criteria were as follows: (1) illiteracy; (2) presence of another neurological disorder; and (3) presence of depression. Drop-out criteria were as follows: (1) change in medicine including acetylcholine esterase inhibitors and NMDA antagonists; (2) participation in other non-pharmacological treatment program(s); (3) attendance of ≤60% in the intervention group; and (4) presence of depression. All participants provided informed consent and this study was approved by Haeundae Paik hospital institutional review board (IRB no. 2018-11-019).

**Figure 1:**
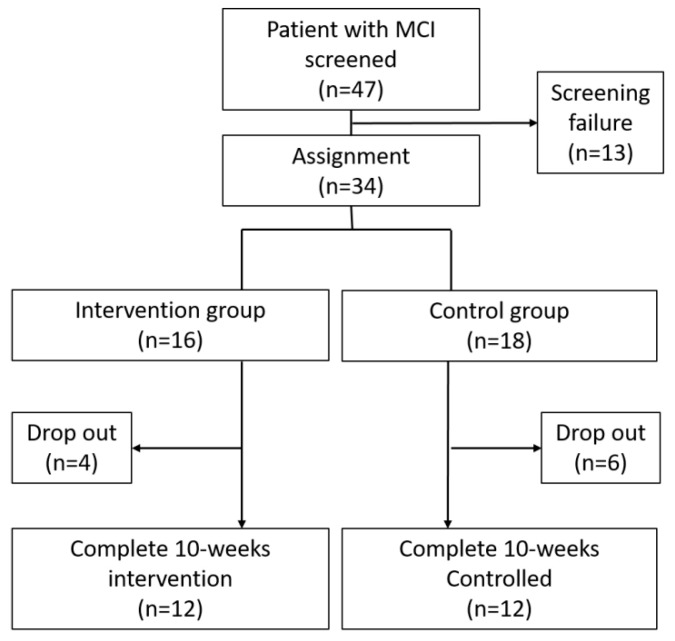
Flowchart showing the process of study enrollment. Forty-seven patients with mild cognitive impairment were recruited for this study and thirty-four patients were selected to be randomly assigned to the intervention (n = 16) and control (n = 18) groups. Four patients in the intervention group and six patients in the control group dropped-out. Finally, 24 patients (12 patients in the intervention group and 12 patients in the control group) completed the study protocol.

The flowchart showing the enrollment process is presented in Figure 1. Forty-seven patients diagnosed with MCI were recruited for our study. After screening, 34 participants were randomly assigned in 1:1 ratio to the intervention and control groups. 16 patients were assigned to the intervention group and 18 patients were assigned to the control group. During the intervention period, four participants dropped-out of the intervention group and six dropped-out of the control group. Two participants (1: intervention group, 1: control group) changed their medication because of disease progression and the remaining eight participants withdrew their consent for participation in this study. Finally, there were 12 patients each in the intervention and control groups.

This study was divided into two phases according to the research goals. First, we developed the digital devices and the protocol of Song-based Cognitive Stimulation Therapy (SongCST) by interdisciplinary approaches from the perspective of neurology, music therapy, computer science, industrial design, and electronic science. Subsequently, we conducted a clinical pilot test to verify the effectiveness of the digital device-generated musical stimuli on the cognitive function of patients with MCI using the protocol of SongCST. The musical stimulus-generating digital devices were composed of percussion- and keyboard-shaped instruments. Playing the instruments involved performing timed and colored visual-motor detection tasks presented with moving lyric patterns on a computer screen based on each tempo (bpm) according to the protocol of SongCST. 

### 2.2. Therapeutic Musical Devices as Cognitive Intervention

#### 2.2.1. Development of Therapeutic Musical Devices

Our multidisciplinary research teams developed the computerized sensory–motor multitask devices that generate musical stimuli [21]. The theoretical frameworks of the digital devices generating musical stimuli were as follows: (1) According to neurocognitive psychology and the aging brain-based music therapy strategies [18], physical musical activities based on human rhythmicity promote the neural circuits in the brain (e.g., certain motor structures such as basal ganglia and supplementary motor areas in the brain appear to be particularly responsive to beat processing)[16]. (2) When the brain recognizes and repeats rhythmic elements of music, related neural circuits are stimulated (e.g., strong beat and similar stimuli do not induce a beat percept at all)[22]. (3) Clinical music cognition research based on synchronized finger or hand tapping revealed an extensive brain network related to prediction during sensorimotor synchronization (SMS) using auditory pacing sequence maintaining tempo changes [23]. (4) It facilitates the storage and retrieval of non-musical information through the consolidation process of encoding, organizing, and elaboration using regular and repetitive patterns of rhythm and lyrics [24].

**Figure 2:**
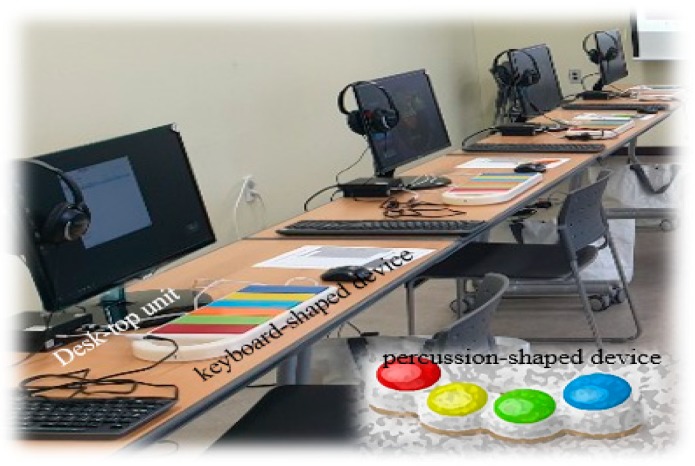
Basic settings of devices. Therapeutic musical devices comprise a desk-top unit, monitor, speaker, headphone, and percussion- and keyboard-shaped electrical devices on the desk.

The digital devices with musical stimuli were composed of a desk-top unit, monitor, and speaker, as well as percussion- and keyboard-shaped devices. The shape of musical devices, color selection based on a clinical survey, and complexities of color matching were determined by a neurologist, music therapist, and industrial design expert considering the visual and cognitive-motor functions affected by MCI as well as the relevance of playing instruments [15]. We developed our devices in collaboration with an electronics company (Ilsan electronics, Korea) and our devices were connected to a 15-inch monitor (Samsung, Korea). The percussion-shaped device had four colors and was played with both the hands and palms. The keyboard-shaped device had six colors so that a patient can play it using fingers of both hands. The therapeutic musical devices included timed and colored visual-motor detection tasks synchronized with the lyric presentation at the tempo of each song on the computer screen (15 inch). Although patients wore individual headphones to listen to the musical stimuli clearly, the speaker was used for sound output. The basic setting of devices is shown in Figure 2.

Patients were asked to tap the musical stimuli-presenting digital devices in synchronization with the color-matching stimuli aligned with the isochronous beats by each song. Auditory and tactile feedbacks, such as a percussion ‘tock’ sound was provided each time the patient tapped [25]. They could listen to each song and auditory feedback only through the headphones, which helped reduce noise from the surrounding environment. The computer algorithm and musical devices were developed considering the sound and visual cue quality, so that the musically timed visuomotor cues and the responses of patients could be recorded exactly in the desk-top unit [26] (Supplementary video 1). Moreover, the individual accuracy score and the response time (sec), such as the inter-tap interval and inter-onset interval for each task, were automatically recorded in the desk-top unit. The researchers monitored and estimated the patient’s performance by checking the data recorded in the devices.

#### 2.2.2. Development of the Song-Based Cognitive Stimulation Therapy (SongCST) Protocol

While we invented the digital devices with musical stimuli, a neurologist and a music therapist developed the therapeutic protocol of music therapy for improving the cognitive function in patients with MCI using our devices. The theoretical frameworks for the protocol of SongCST are as follows: (1) Appropriate acoustic and temporal factors of songs that can stimulate the auditory-visual stimuli in the brain are critical for the proper entrainment of patients to respond to the task provided [22]. (2) The musical components of songs, such as specific tempo (e.g., bpm and duple or triple meter), rhythm (e.g., simple, complex, syncopated rhythm, or non-beat based rhythm), melody (e.g., various intervals, tonal, musical mode, or voice parts), lyrics (e.g., specific letter patterns, related emotion, or memory), forms (e.g., repeated musical phrases, specific phrases, or various accompaniment’s styles), and so on, may help different integrated sensory-motor responses through listening to music and playing the instruments on the basis of external musical cues [27]. (3) The period of entrainment helps regulate and synchronize the rhythmic cue to optimize movement patterns and performance of timed visuomotor tasks [25]. (4) The lyrics and musical traits of songs provided not only emotional reminiscence and pleasure, but also predictable external cue as structured auditory stimuli [28]. These may provide the emotional and cognitive support needed by patients with MCI to focus on consecutive multitasking while using these devices [29].

**Figure 3:**
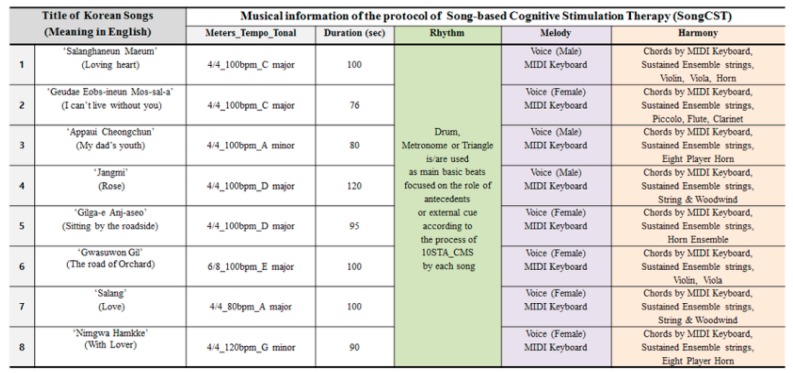
Musical information on eight songs. Musical information and components of each song, considering the therapeutic musical elements of acoustic, cognitive, and emotional function of elderly patients with MCI.

The following process was used to develop the SongCST protocol. First, the certified music therapist selected therapeutic functional songs for our musical devices considering the musical elements of acoustic, cognitive, and emotional functions for elderly patients with MCI [14,28]. The selected Korean popular songs had steady beats, positive lyrics, and simple and repeated musical patterns for elderly patients [24]. The musical information of each song is presented in Figure 3. Second, the music therapist created the 10 stages of therapeutic arrangements for cognitive motor stimulation (10STA_CMS), with each original song, considering therapeutic musical components such as rhythm, melody, harmony, meters, tempo, tone, and so on [30]. Then, the music therapist recorded the selected songs based on the 10STA_CMS so that these music files were mounted in our desk-top unit as external musical stimuli [16]. The protocol of SongCST content validation was checked by three certified music therapists with 200–1000 hours of clinical experience within a geriatric clinical setting.

**Figure 4:**
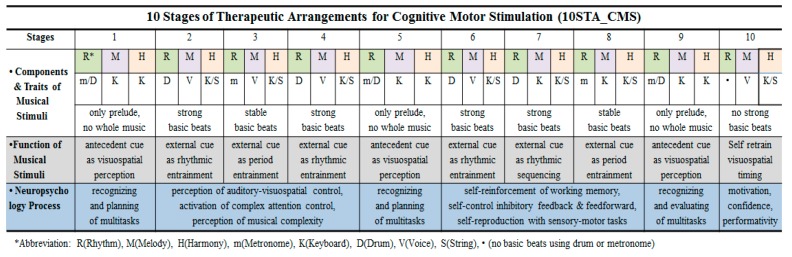
Therapeutic process and functions of 10STA_CMS based on modulated rhythm, melody, and harmony. This figure includes musical traits, functions, and the specific neurocognitive process of musical stimuli as external cues with therapeutic musical devices.

The components and traits of 10STA_CMS, with/without steady beats with drum or metronome, melody with a keyboard or singer’s voice with lyrics, simple chord progressions and specific accompaniment styles stimulated and modulated the neural network for the elderly patients with MCI [31], may help the functions of auditory-visuo-spatial stimulus, complex attention control, executive function, working memory, eye-hand coordination, immediate reward, entrainment and inhibitory control, feedback and feedforward interaction, and spontaneous tactile stimuli [17,23]. The detailed therapeutic process and functions of 10STA_CMS are presented in Figure 4.

### 2.3. Group Music Therapy Administered Using Digital Device-Generated Musical Stimuli

The planned sequence of the group music therapy program using the digital devices with musical stimuli consisted of four phases based on therapeutic mechanisms of music therapy [18] and neurocognitive psychology [30]. During the ten-week intervention, a total of eight songs were provided each week according to the following therapeutic phase plan.

**Phase 1. Introduction of activity** (1st–4th sessions): In the ‘attention’ phase, patients were provided instructions for performance multitasking using musical devices in a group. The music therapist provided the protocol of SongCST or no musical stimuli individually to evaluate each patient’s physical, cognitive, and emotional function as part of a music therapy assessment. 

**Phase 2. Adaptation of the digital devices** (5th–10th sessions): In the ‘relevance’ phase, patients were adapted to participate in various tasks such as timed color matching tasks, different visuospatial tasks using two musical devices, targeted lyrics pattern for matching tasks, operation system of devices, complexity of tasks according to each song (list #1–#4) and complexity of 10STA_CMS in a group or individual setting. 

**Phase 3. Concentration on activities by self** (11th–16th sessions): In the ‘confidence’ phase, patients voluntarily concentrated on various changes such as timed matching tasks, complexities of tasks, hand/finger movements, and repeated lyrics patterns based on each song (list #3–#8) in an individual setting using headphones. 

**Phase 4. Reinforcement in activity** (17th–20th sessions): In the ‘satisfaction’ phase, patients experienced self-reinforcement for more musical participation such as group playing, self-selection of all songs (list #1–#8) and tasks, and playing percussion instruments in a group or individual setting.

**Figure 5:**
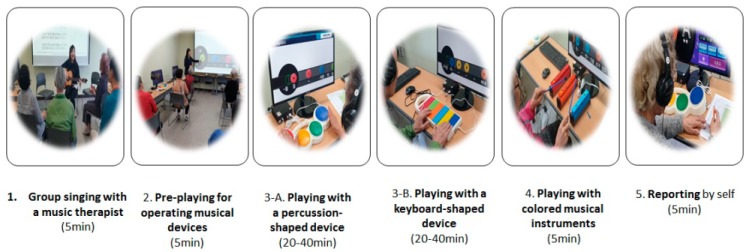
Five progressive steps of each session. Constructive processes included were group singing with a music therapist, pre-playing in a group, playing with therapeutic musical devices as therapeutic cognitive intervention, and self-reporting of experiences of each session.

All patients sat at their desks and wore headphones individually to perform and concentrate on musical tasks by manipulating the protocol of SongCST on the devices. However, overall cognitive interventions were performed as group therapy because participants communicated with each other and shared the contents and guidance provided by the music therapist. The music therapist checked the sound of the musical devices as well as the level of the individual process considering patients’ performance. In detail, each session of group music therapy program consisted of five progressive steps as shown in Figure 5.

### 2.4. Outcome Measurement

The primary endpoint was the evaluation of the effect of music therapy using digital devices with the SongCST protocol on the patients’ cognition. To facilitate the estimation of the cognitive function, all participants underwent the Mini-Mental State Examination-Dementia Screening (MMSE-DS), Korean Montreal Cognitive Assessment (MOCA-CK), and Clinical Dementia Scale Sum of Boxes (CDR-SOB). We compared the MMSE-K and MOCA-K scores at baseline with those after a 10-week intervention. We also compared the rate of score change between the intervention and control groups from baseline to the end of the 10-week intervention period. Our program was designed to enhance the executive function; therefore, we compared sub-item scores of MOCA-K, which reflect the frontal lobe function, at baseline with those after a 10-week intervention. The secondary endpoint involved the evaluation of the effect of the intervention with the protocol of SongCST on the depressive mood, severity of dementia, and quality of life. To determine the secondary endpoint, we used Beck Depression Inventory (BDI) and the Quality of Life—Alzheimer Disease questionnaire (QOL-AD).

### 2.5. Statistical Analysis

We used SPSS version 18 for statistical analysis (IBM Corp., Armonk, NY). Categorical variables in the demographic data were analyzed by chi-squared test and Fisher’s exact test. For the comparison of scores between time points, we used paired *t*-test and Wilcoxon signed rank test. For the comparison of differences between the intervention and control group after the 10-week intervention period, relative to baseline, we used independent *t*-tests.

## 3. Results

Demographic information and baseline cognitive function scores of all the participants are shown in Table 1. In our study, 13 men (intervention group, five; control group, eight) and 11 women (intervention group, seven; control group, four) were enrolled. Age, sex, education level, and history of AchEi use were not significantly different between the groups. Baseline MMSE-DS, MOCA-K, CDR-SB, BDI, and QOL-AD scores were also not significantly different between the intervention and control groups.

Table 2 shows the difference between clinical scores at baseline and the 10-week intervention time point and their significance levels as determined by paired *t*-test. MMSE-DS scores were significantly higher after 10-week intervention than at baseline (*p* = 0.002) in the intervention group. MOCA-K scores and CDR-SB were also significantly higher after 10 weeks of training than at baseline (*p* = 0.046, *p* = 0.05, respectively). However, there were no significant changes in BDI and QOL-AD scores after intervention. In the control group, there were no significant differences in MMSE-DS, MOCA-K, BDI, CDR-SB, and QOL-AD scores at different time points.

Table 2 also showed the comparison of mean changes in clinical scores between intervention and control groups. The mean change in MOCA-K scores after 10 weeks is 2.33 ± 3.60 in the intervention group and −1.00 ± 3.62 in the control group, and the change was significantly different between the groups (*p* = 0.034). The mean changes in CDR-SB also showed significant difference between the intervention and control groups (−0.25 ± 0.40 vs. 0.25 ± 0.87; *p* = 0.013). However, the changes in MMSE-DS, BDI, and QOL-AD scores were not significantly different. MMSE-DS score, which showed significant improvement within the intervention group, did not show a significant difference between the groups.

Analysis of sub-item scores of MOCA-K are shown in Table 3. Executive/visuospatial function (3.83 ± 1.20 vs. 3.92 ± 0.10; *p* = 0.00), sentence making (2.50 ± 0.67 vs. 2.58 ± 0.51; *p* = 0.02), delayed memory (1.58 ± 1.44 vs. 2.17 ± 1.85; *p* = 0.00), and orientation (5.08 ± 0.90 vs. 5.50 ± 0.52; *p* = 0.01) showed significant improvement after intervention. However, world fluency, attention, and abstract thinking showed no significant improvement after the intervention.

## 4. Discussion

Our study evaluated the effect of a cognitive intervention involving digital devices that generate musical stimuli with the SongCST and 10STA_CMS protocols in a group music therapy program. The results demonstrated that the intervention showed a positive effect on the cognitive function of patients with MCI. Our multidisciplinary approach considered the perspectives of a clinician, musical therapist, industrial designer, and computer scientist for the elderly [32].

Similar to patients in the intervention group, participants in the control group also showed an increase in cognitive scores after 10 weeks; however, in the latter case, the increase was statistically insignificant. For preventive purposes, about 30% of the participants in both groups used AchEi; therefore, it might have affected the overall cognition. MMSE-DS scores were higher in the intervention group than the control group. However, all the participants with MCI had different baseline MMSE scores, and therefore, we compare the rate of change of each score. Thus, the influence of this bias on our results is low.

Many reports on the application of music therapy for dementia have been published; however, the results were inconsistent [12,13]. Most studies showed an improvement in memory and an increase in MMSE scores [33]. Our results are in line with these results; our study also shows that MOCA-K scores, which can reflect the frontal lobe function, are significantly higher than the scores of other scales and that this difference is higher in the intervention group than in the control group [34,35]. Sub-item analysis of MOCA-K scores showed that the improvement in the executive function and delayed memory was more prominent than that in other domains. Hand-eye coordination guided by auditory stimuli improved the executive and visuospatial function as well as memory. Moreover, multiple steps depending on difficulty level with the complexities of musical tasks and cognitive-motor function might be effective in the frontal lobe function [25].

In the seven sub-item analyses of executive function on the MOCA-K scale, ‘attention’ was evaluated on the basis of patients’ ability to read a list of digits, tapping with specific letters, and subtract seven from 100. Attention showed no statistically significant improvement. ‘Attention’ is crucial for musical cognition; it facilitates the recognition of similarity, proximity, and complexity of musical stimuli. Our research indicated that slightly different levels of information are processed in the frontal lobe of patients with MCI, which ranges between the ‘attention’ function of MOCA-K and the ‘attention’ function of the multisensory-motor musical task in this study [21,23,34]. For example, the Sound Training for Attention and Memory (STAM-Dem) protocol is designed for patients with neurodegenerative impairment to facilitate cognitive rehabilitation and to investigate the patients’ musical relationship [36]. Therefore, in terms of ‘attention’ evaluation, it is necessary to provide intensive music therapy focused on specific attention functions and to use an objective evaluation tool that reflects the specific characteristic effects of musical stimuli on patients rather than general music and paper-based evaluation methods.

We used CDR-SB scores for estimating the severity of cognitive impairment. The intervention group showed a significantly higher CDR-SB score after intervention than the control group did, relative to that at baseline. Our results are similar to those published in a previous report, which demonstrated the adjuvant effect of music therapy in patients with AD [37]. This report showed that CDR-SB scores were significantly higher after music therapy than the scale scores including those of MMSE, neuropsychiatric inventory, and cognitive abilities screening instrument. QOL-AD and depression scores did not improve after treatment. Our music therapy program contains repetitive and well-structured processes and requires more multisensory-motor coordination, program-focused repetition, and engaging CT than social and psychological musical activities for emotional regulation [14,38]. Our program was schedule-focused, and it enhanced the executive function more than mood and depression.

Music therapy is not commonly applied in a clinical setting as non-pharmacological treatment despite increasing positive evidence [34,39]. However, our results demonstrate that cognitive intervention with music stimuli can be operationalized to improve the cognitive function of patients with MCI. Before the musical cognitive intervention, a pre-test with nine participants for evaluating the effectiveness of music therapy was performed. The results of the pre-test showed that the musical stimuli dramatically increased the accuracy of performance with musical devices than without musical stimuli. We assumed that music stimuli contributed to the improvement of cognition by increasing compliance and concentration on the intervention [29].

Currently, many patients with cognitive impairment are not receiving adequate care and non-pharmacological treatment because of an increasing care-giver burden and cost of non-pharmacological treatments despite a lot of systemic effort. Digital music devices can address the current problem of cost by computerizing individual CT programs and making them suitable for use at homes, nursing homes, or clinics [40]. A previous report showed the positive effect of a simple ‘tone chime’-like musical instrument on the cognition of patients with MCI [41]. By contrast, our devices, designed on the basis of research focused on geriatrics [42], integrated real musical devices, high-quality training content, and a design suitable for the elderly. Consequently, this study can enable individualization of Information Technology (IT) and music therapy to reduce the risk and impact of dementia using personal electronic instruments. 

Music therapy research on preventing dementia symptoms often lacks interventional details and researchers may not adequately consider the complexity of musical tasks and the cognitive complexity required for managing/preventing MCI [43,44]. We attempted to address this by engaging an interdisciplinary research team. The protocol of SongCST was co-developed by a clinician and a certified music therapist specializing in neurodegenerative diseases. This protocol focuses on the levels of cognitive-motor function required for elderly patients to play musical instruments in terms of tempo, rhythm pattern, and change in complexity of sensory-motor multitask with songs as well as emotional reminiscence and engagement of vocal accompaniments of each song. Additionally, evidence-based musical instrument playing methods such as the steady and predictable musical time cues, sequencing and spatial organization [16,21,45], and sub-components of tasks that stimulate the related neural circuits in the brain were also considered [15,17,23].

This study provides evidence for the possibility of employing feedback and a monitoring system of digitalized music therapy such as the curative algorithmic music [46]. Personal information and performance data were stored in the desk-top unit cumulatively. This allows clinicians and music therapists to plan individual protocols and start therapeutic musical activities [47,48]. This study takes a step toward precision medicine that is beyond hospital-based treatment [49]. Moreover, it can make computerized individual CT programs with musical activities possible for future smart homes or hospitals’ internet of things (IOT) systems by allowing certified music therapists and clinicians to provide and monitor ambient assisted living (AAL) and ubiquitous-health (U-health) care systems for the elderly.

There were several limitations to this study. First, this is pilot study; therefore, the sample size is small. However, we did reach statistically meaningful results. Secondly, we could not perform more detailed neuropsychological tests such as the ADAS-cog test for evaluating cognitive function. However, our study did show meaningful improvement in MOCA and CDR-SB scores, which reflect cognitive function and disease severity.

## 5. Conclusions

Cognitive interventions with musical stimuli generated by digital devices made by a multidisciplinary team have significant effects in the cognitive function of patients with MCI. Song-based cognitive stimulation with this device for 10 weeks improved MOCA-K and CDR-SB scores. Our study demonstrated that music therapy using digitalized devices has positive effects on the cognition of patients with MCI.

## Figures and Tables

**Table 1:** Demographic data.

	Intervention Group (n = 12)	Control Group (n = 12)	*p*
Sex (M/F)	5/7	8/4	0.22^1^
Age (years)	72.83 ± 7.93	73.42 ± 5.96	0.81^2^
Education (years)	8.33 ± 2.81	10.17 ± 3.13	0.14^2^
MMSE-DS	24.92 ± 1.51	23.33 ± 2.10	0.05^2^
MOCA-K	21.42 ± 3.96	20.67 ± 3.60	0.63^2^
CDR-SB	1.63 ± 0.64	1.75 ± 1.06	0.73^2^
BDI	13.42 ± 7.55	12.42 ± 8.56	0.76^2^
QOL-AD	30.33 ± 6.04	28.27 ± 4.82	0.38^2^
AchEi use	4/12 (33.33%)	3/12 (25%)	1.00^3^

^1^*p* values were derived from chi-squared distribution. ^2^ *p* values were derived from independent *t*-test. ^3^ *p* values were derived from Fisher’s exact test. MMSE-DS: Mini-Mental State Examination-Dementia Screening, MOCA-K: Montreal Cognitive assessment-Korean, BDI: Beck depression inventory, CDR-SB: Clinical Dementia Rating- Sum of Box, QOL-AD: Quality of Life-Alzheimer Disease, AchEi: Acetylcholine esterase inhibitor.

**Table 2:** The comparison of clinical outcomes between baseline and post-intervention in both the groups.

	Intervention Group (n = 12)				Control Group (n = 12)				*p*
	Baseline	10 Weeks	Difference	*p*	Baseline	10 Weeks	Difference	*p*	
MMSE-DS	24.92 ± 1.51	26.83 ± 1.80	1.92 ± 1.62	0.002^1^*	23.33 ± 2.10	24.17 ± 2.55	0.83 ± 1.95	0.166^1^	0.153^3^
MOCA-K	21.42 ± 3.96	23.75 ± 2.56	2.33 ± 3.60	0.046^1^*	20.67 ± 3.60	19.67 ± 3.11	−1.00 ± 3.62	0.359^1^	0.034^3^*
BDI	13.42 ± 7.55	14.50 ± 9.89	1.08 ± 6.11	0.552^1^	12.42 ± 8.56	10.00 ± 8.47	−2.42 ± 8.88	0.366^1^	0.273^3^
CDR-SB	1.63 ± 0.64	1.38 ± 0.71	−0.25 ± 0.40	0.053^1^	1.75 ± 1.06	2.00 ± 0.88	0.25 ± 0.87	0.233^2^	0.013^4*^
QOL-AD	30.33 ± 6.04	31.42 ± 5.79	1.08 ± 3.32	0.282^1^	28.27 ± 4.82	29.00 ± 4.07	0.82 ± 4.60	0.568	0.875

^a1^*p* values were derived from paired *t*-test. ^2^ *p* values were derived from Wilcoxon signed rank test. ^3^ *p* values were derived from independent *t*-test. ^4^ *p* values were derived from Mann-Whitney’s U test. Shapiro–Wilk’s test was employed for test of normality assumption.

**Table 3:** Sub-item analysis of MOCA-K scores in the intervention group.

Function	MOCA-K		
	Baseline Score	Score after 10 Weeks	*p*-Value
Executive/visuospatial	3.83 ± 1.20	3.92 ± 0.10	0.00*
Word fluency	2.50 ± 0.52	2.83 ± 0.39	0.14
Attention	4.50 ± 1.24	4.92 ± 1.08	0.46
Sentence	2.50 ± 0.67	2.58 ± 0.51	0.02*
Abstract thinking	1.83 ± 0.39	1.42 ± 0.67	0.35
Delayed memory	1.58 ± 1.44	2.17 ± 1.85	0.00*
Orientation	5.08 ± 0.90	5.50 ± 0.52	0.01*
			*p* < 0.05*

^1^*p* values were derived from paired *t*-test.

## Supplementary Materials

The following are available online at https://www.mdpi.com/2227-9032/8/1/45/s1.
